# Serum vitamin D deficiency and risk of epithelial ovarian cancer in Lagos, Nigeria

**DOI:** 10.3332/ecancer.2020.1078

**Published:** 2020-07-23

**Authors:** Emmanuel Adekunle Sajo, Kehinde Sharafadeen Okunade, Gbenga Olorunfemi, Kabiru Afolarin Rabiu, Rose Ihuoma Anorlu

**Affiliations:** 1 Department of Obstetrics and Gynaecology, Lagos University Teaching Hospital LUTH, PMB 12003, Idi-Araba, Lagos, Nigeria; 2 Department of Obstetrics and Gynaecology, Faculty of Clinical Sciences, College of Medicine, University of Lagos, PMB 12003, Idi-Araba, Lagos, Nigeria; 3 Division of Epidemiology and Biostatistics, School of Public Health, University of the Witwatersrand, Parktown 2193, Johannesburg, South Africa; 4 Department of Obstetrics and Gynaecology, Lagos State University College of Medicine, PMB 21266, Ikeja, Lagos, Nigeria

**Keywords:** CALBIOTECH®, EOC, Lagos, Nigeria, ovarian cancer, vitamin D

## Abstract

The studies that have evaluated the association between vitamin D and risk of ovarian cancer have reported inconsistent findings. Many of these studies were carried out in regions with relatively low sunshine all year round unlike in Africa. This study was aimed to determine the relationship between vitamin D deficiency and epithelial ovarian cancer (EOC) amongst women in Lagos, Nigeria. We conducted a case–control study involving women with histologically confirmed EOC (case group) and an equal number of healthy women without cancer (control group) treated at the gynaecological oncology units of two public tertiary hospitals in Lagos, Nigeria, between 1 August, 2016 and 31 May, 2017. Relevant information was obtained from the participants using a structured interviewer-administered questionnaire, and then, venous blood samples were collected and analysed for serum 25-hydroxyvitamin D levels using the CALBIOTECH® 25(OH) vitamin D ELISA kit. The descriptive statistics were conducted for all relevant data, and the multivariable analysis using binary logistic regression model was performed to examine the association between vitamin D deficiency and EOC after adjusting for all possible confounders. The mean age of the participants was 50.6 ± 11.1 years. There was no statistically significant association between serum vitamin D deficiency and EOC (*p* = 0.09). However, 10 mmol/L change in circulating vitamin D levels was associated with EOC amongst the study participants (adjusted odds ratio 0.96; 95% confidence interval 0.93–0.99; *p* = 0.04), but following adjustment for potential confounders in a multivariable analysis, there was no statistically significant relationship observed with EOC (adjusted odds ratio 0.99; 95% confidence interval 0.97–1.00; *p* = 0.06). In addition, there was no evidence of an interaction effect between these confounders and change in circulating 25(OH)D levels in relation to the risk of EOC. The study revealed no statistically significant association between the circulating levels of vitamin D and the risk of EOC. A better assessment of sun exposure in the future as well as better dietary compositional data may help to clarify whether the association between vitamin D and EOC actually exists. Therefore, the future large prospective longitudinal studies are recommended to further examine this relationship and then evaluate the possible need for vitamin D supplementation in women with an increased risk of EOC in Nigeria.

## Introduction

Ovarian cancer is a very deadly cancer, and it is the second most common cause of gynaecological cancer death in Nigeria [1]. Over 70% of ovarian cancer patients present at the late stage of the disease when little or nothing could be done, thereby resulting in poor prognosis and survival [2]. In Nigeria, epithelial ovarian cancer (EOC) accounts for 60%–68% of all histological types of ovarian cancer [2–4]. Unlike the most prevalent cancer, cervical cancer, which has preventive and screening strategies, there has not been any preventive modality nor validated screening programme for ovarian cancer as most of the risk factors are not modifiable [2].

Ovarian cancer incidence is now rising in Nigeria and most low- and middle-income countries due to the gradual transition to a westernised way of life [5], and this will impact significantly on the already overstretched healthcare system in these counties. Several hereditary/genetic, reproductive, nutritional and environmental risk factors have been directly or indirectly linked to the development of ovarian cancer. Risk estimates are mostly based on a family history with a lifetime risk estimate of 2–5 times the population risk for individuals who have one first degree relative with ovarian cancer [6, 7]. Reproductive factors such as early age at menarche and late age at menopause, which increase the risk of ovarian cancer, have also been shown to modestly increase the risk of ovarian cancer [8]. Several protective factors have been established in the previous studies, and these include increasing parity and breastfeeding [9], oral contraceptives [10] and increased physical activities [11]. The process of cellular inflammation, wound healing and hormonal influence are all thought to be involved in the poorly understood ovarian cancer pathogenesis [12].

In the medical world, there is an increased interest in the diverse functions of vitamin D in the human body [13, 14]. Vitamin D, a fat-soluble vitamin, which is mostly produced from sunlight exposure [15, 16], has been found in experimental studies to inhibit the growth of ovarian cancer cells and also stimulate programmed cell death in similar cells. The circulating form of vitamin D, 25-hydroxyvitamin D (25[OH]D), is converted to 1,25-dihydroxy vitamin D (1,25[OH]­­­_2_D by 1-α-hydroxylase enzyme which is ubiquitous in most tissues of the body. Both normal and cancerous ovarian cells have vitamin D receptors, through which the active form of vitamin D acts as a paracrine hormone to cause antiproliferative action through the non-calcaemic pathway. It probably reduces the risk of cancers by the induction of tumour suppressor genes such as p53, p21 and DNA-mismatched repair genes [17].

There are no reliable data on the intake of vitamin D-fortified diet amongst Nigerians, and it is difficult to study the dietary intake of vitamin D as it is strongly associated with lactose intake [18, 19]. Thus, circulating vitamin D levels may be a useful alternative in investigating a potential relationship with ovarian cancer. However, to the best of authors’ knowledge, no study has examined the association between low circulating vitamin D levels and the risk of ovarian cancer in Sub-Saharan African women. The studies that evaluated this association reported inconsistent findings [17, 20, 21], and many of these studies were conducted in regions with a predominantly low prevalence of micronutrient deficiencies, unlike what is obtained in most of the resource-constraint settings including Nigeria. This study, therefore, aimed to determine the association between serum vitamin D deficiency and the risk of EOC amongst women in Lagos, Nigeria. The finding of this study may give an insight into the future preventive role of this important vitamin against this extremely lethal female gynaecological cancer.

## Materials and methods

### Study design and setting

This was a case–control study carried out at the gynaecological wards of the Lagos University Teaching Hospital (LUTH) and Lagos State University Teaching Hospital (LASUTH) between August 1, 2016 and May 31, 2017. LUTH and LASUTH are the two main public tertiary institutions that offer specialised cancer care in Lagos, Nigeria. Lagos State is located in the Southwestern part of Nigeria, Coordinates: 6°35′N 3°45′E; Land Area—3,474 km^2^ (1,341 sq. mL).

### Study population and eligibility criteria

The study enrolled two groups of women by consecutive sampling technique. The case group included women who had staging laparotomy and histologic confirmation of epithelial ovarian cancer (EOC), whereas their control group comprised of age-matched (±5 years) women attending the gynaecology outpatient clinics of the two hospitals on account of infertility and had no clinical or ultrasound scan evidence of ovarian lesion. Women who used vitamin D-containing mineral supplements in the past 3 months before enrollment and those who refused consent at enrolment or withdrew their consents in the course of the study were excluded from the study.

### Sample size calculation

The sample size was determined by applying the formula for a comparison of two means using data from the published study by Webb *et al* [22]. With a standard deviation of 18.7 nmol/l and between-group vitamin D level mean difference of 15 nmol/L, a power analysis revealed that a minimum sample size of 35 women in each group (total sample size of 70 women) is required to ensure at least 80% power for detecting the anticipated between-group differences and to compensate for a non-response rate of 10%. We enrolled 35 women with histologically confirmed EOC and 35 cancer-free comparison groups into the study.

### Data collection

A structured interviewer-administered questionnaire was used to collect data such as sociodemographic characteristics, medical history, family history of cancer, smoking as well as information on the use of vitamin D supplements and dressing style (whether covered or uncovered). The covered dressing was defined as the covering of the entire body except for the hands and feet, whereas the uncovered dressing was the common dressing with exposure of the face, neck, hand and feet. The participant’s skin colour was also recorded as evident from the face, neck, arms and legs. The anthropometric measurements such as weight and height were determined, and the body mass index (BMI) was calculated for each participant. A venous blood sample (4 mL) was collected from each participant and sent to the laboratory. The samples were then centrifuged, and the serum was stored at −20°C until analysis. The level of total 25-hydroxyvitamin D in each serum sample was measured by using a solid-phase competitive enzyme-linked immunosorbent assay (ELISA) as per the manufacturer’s instructions (Calbiotech®, Spring Valley, CA, USA). The specificity of the assay for measurement of total 25 (OH) vitamin D was 100% with an intraassay variation of <6% [23]. Low serum vitamin D was defined as a level below 75 nmol/L [15].

### Statistical analysis

The statistical analysis was conducted using Stata Version 16 Statistical Package (Stata I/C, StataCorp LP, Texas, USA). The descriptive statistics were computed for all relevant data. Categorical variables were described as frequency and percentages. Normally distributed continuous variables were described as mean ± standard deviation, whereas non-normally distributed continuous variable was described as median and interquartile range. The Kolmogorov–Smirnov test was used to test for normality. The independent sample *t*-test or Mann–Whitney U-test was used to compare the continuous variables amongst participants with ovarian cancer and those without ovarian cancer. The Pearson’s Chi-square test (or Fischer’s exact test) was used to test the association between categorical variables and EOC. The univariable and multivariable conditional logistic regression modellings were conducted. The model building utilised variables based on the authors’ clinical knowledge and biological plausibility. The potential confounders of EOC and circulating vitamin D levels, specifically participants’ age, parity, menopausal status, body mass index, family history of cancer, educational qualification, skin complexion and coexisting medical morbidity were considered in the logistic regression models. The participants’ age and variables with *p-*value < 0.20 such as a family history of cancer and skin colour were built into the final multivariable conditional logistic regression model using vitamin D as a continuous primary explanatory variable. Subsequently, we estimated the main and circulating vitamin D level covariate interaction effects on the risk of EOC. A two-tailed test of hypothesis was assumed, and the *p*-value < 0.05 was considered to be statistically significant.

### Ethical considerations

An ethical approval for the study was obtained from the Health Research Ethics Committee of the two participating teaching hospitals before the recruitment of participants. All participants were counselled before enrolment and read and signed an informed consent form. The investigators ensured strict confidentiality of participants’ information.

## Results

**Figure 1.**
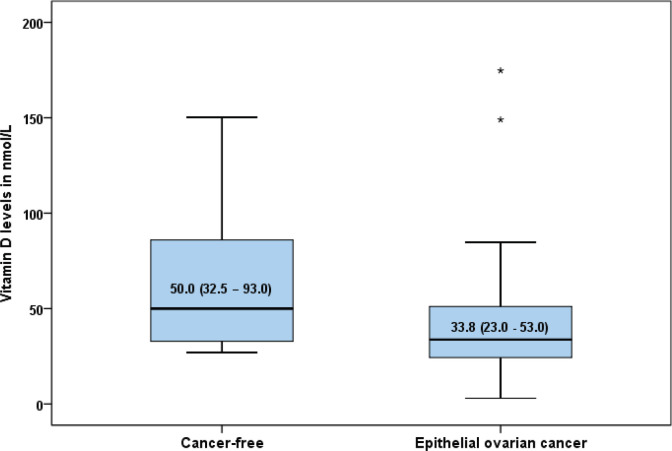
Box plot showing the median serum vitamin D levels in women with epithelial ovarian cancer and those without cancer (50.0 (32.5–93.0) versus 33.8 (23.0–53.0) nmol/l; *p* = 0.005).

The characteristics of the study women in each group are shown in Table 1. The mean age was 50.46 ± 11.68 years for women with epithelial ovarian cancer (EOC) and 50.40 ±11.10 years for those without cancer (*p* = 0.47). There were no statistically significant differences in the parity (*p* = 0.83), occupation (*p* = 0.99), educational level (*p* = 0.36), menopausal status (*p* = 0.63), BMI (*p* = 0.73), skin colour (*p* = 0.37), alcoholic beverage ingestion (*p* = 0.61) and family history of cancer (*p* = 0.04) between the EOC group and their cancer-free comparison group. The overall prevalence of vitamin D deficiency was 77.5% (*n* = 54) with 85.7% (*n* = 30) of women with EOC having low serum vitamin D level compared to 68.5% (*n* = 24) of women without EOC (*p* = 0.09). The median (interquartile range) level of vitamin D was 33.8 (23.0–53.0 nmol/L) in the EOC group and 50.0 (32.5–93.0 nmol/L) in the cancer-free group (*p* < 0.01) (Fig. 1).

The most common histological type of EOC in the study was serous cystadenocarcinoma (80.0%), whereas the least common was malignant Brenner tumour (2.9%). Majority of the women had elevated serum cancer antigen 125 (91.4%) and presented with an advanced stage of the disease (94.3%) (Table 2).

Table 3 shows no statistically significant association between the categories of vitamin D (<75 nmol/L versus ≥75 nmol/L) and EOC (adjusted odds ratio 2.75; 95% confidence interval 0.84–9.00; *p* = 0.09). However, there was a statistically significant association between 10 mmol/L change in circulating serum vitamin D levels and EOC after a univariable analysis (adjusted odds ratio 0.96; 95% confidence interval 0.93–0.99; *p =* 0.04). With further multivariable conditional logistic regression analysis after adjusting for the potential major risk factors of EOC such as age, family history of cancer and skin colour, the change in circulating serum vitamin D levels was found to have no statistically significant association with EOC (adjusted odds ratio 0.99; 95% confidence interval 0.97–1.00; *p* = 0.06). However, there was an almost 6-fold statistically significant association between the patients’ family history of cancer and the risk of EOC (adjusted odds ratio 5.88; 95% confidence interval 1.05–32.94; *p* = 0.04). In addition, there was no evidence of an interaction effect between age, family history of cancer or skin colour and circulating 25(OH)D levels in relation to the risk of EOC (Table 4).

## Discussion

This study showed no association between circulating vitamin D levels and epithelial ovarian cancer (EOC). Initial support for a protective role for vitamin D in ovarian cancer was provided by previous ecological studies that recorded inverse associations between ultraviolet-B (UV-B) irradiance exposure from sunlight and ovarian cancer incidence or mortality rates [24–27]. However, a few recent studies that evaluated this association further have reported conflicting findings [17, 20, 21]. This study reported similar findings to the studies conducted by Tworoger *et al* [20], Arslan *et al* [17] and Zheng *et al* [28], where no significant associations were detected between circulating serum 25-hydroxyvitamin D levels and the risk of ovarian cancer. However, it appears that Arslan *et al* [17] did not find any significant difference in the median serum 25-hydroxyvitamin D levels between the case group with EOC and their healthy comparison group. On the contrary to the finding, Bakhru *et al* [21] in a similar study conducted in Michigan, USA, a non-tropical country in North America, reported a significant association between low vitamin D levels and ovarian cancer. Bakhru *et al* [21] reported that participants with ovarian cancer had a 3-fold increased risk of having low levels of vitamin D when compared to their counterparts without ovarian cancer. The findings by Toriola *et al* [29] also suggested an increased risk of ovarian cancer in women with low-to-insufficient serum 25(OH)D concentration, thus suggesting that healthy women are usually exposed to sunlight, the major source of naturally occurring vitamin D, all year round [30, 31] as they are mobile and outgoing compared to their counterparts with ovarian cancer who are usually docile and tend to stay indoors all day as a result of their illness. However, the absence of an association between low circulating serum vitamin D levels reported in this present study might reflect the predominantly high prevalence of nutritional deficiencies in this region, but this will require further validation in a more robust longitudinal study that will also objectively assess the diets of these women.

We also recorded in this study that women with a family history of any form of cancer had about 6-fold increased odds of EOC compared to women with no family history of cancer. This was similar to the findings by Tworoger [20] in Boston, Massachusetts, in 2007 and Zheng et al in Nashville, Tennessee, in 2010 [29]. This further suggests that there is a genetic link in the evolution of ovarian cancer as was previously described for women with the BRCA gene mutation [32].

The major limitation of this study was the difficulty in extracting reliable information on the intake of vitamin D-rich diets and vitamin D supplements from some of the study participants, and this might have had an indirect influence on the findings of this study. There is unrestricted access to medications in the country, and most of the people use varieties of supplements purchased over the counter, which may contain different elements including vitamin D. In addition, the study was conducted in the Lagos metropolis inhabited predominantly by well-educated populace within the middle-to-upper socioeconomic status who are expected to take diets rich in most of the nutrients including vitamin D. Moreover, few women in this study had circulating vitamin D levels above 75 nmol/L (22.5%), and thus, we cannot rule out the possibility that women with very high 25(OH)D levels may have a reduced risk of EOC. Finally, the study design type may make it difficult to confirm temporality or causality of the vitamin D exposure as the association observed may be due to changes in the circulating vitamin D levels before the disease due to lifestyle or as a result of prolonged confinement due to illness. Nonetheless, this study was an exploratory and hypothesis-generating project, to serve as the basis for the conduct of a more robust study in the near future. The strength of this study is that it is the first of such, to the best of authors’ knowledge, which will examine the association between low circulating vitamin D levels and the risk of epithelial ovarian cancer amongst women in an Sub-Saharan African region.

## Conclusions

Overall, we observed no clear association between serum vitamin D deficiency and ovarian cancer risk in this study. A better assessment of sun exposure in the future as well as better dietary compositional data may help to clarify whether the association between vitamin D and epithelial ovarian cancer actually exists. Therefore, future large prospective longitudinal studies are recommended to further examine this relationship and then evaluate the possible need for vitamin D supplementation in women with an increased or familial risk of epithelial ovarian cancer in Nigeria.

## Conflicts of interest

The authors declare that they have no conflicts of interest.

## Figures and Tables

**Table 1.** Baseline characteristics of participants^a^.

Characteristics	Epithelial ovarian cancer		*p*-value
	Yes, *n* = 35 (%)	No, *n* = 35 (%)	
**Age in years**			0.47
<50	15 (42.9)	18 (51.4)	
≥50	20 (57.1)	17 (48.6)	
**Mean age ± SD**	50.40 ± 11.68	50.40 ± 11.10	
**Parity**			0.83
0	6 (17.1)	5 (14.3)	
1–4	22 (62.9)	21 (60.1)	
>4	7 (20.0)	9 (25.7)	
**Median parity (IQR)**	3 (1 – 4)	3 (1–5)	
**Occupation**			0.99
Civil servant/retiree	12 (34.3)	11 (31.4)	
Caterer/farmer/housewife	5 (14.3)	6 (17.1)	
Trading	18 (51.4)	18 (51.4)	
**Educational level**			0.36
None/primary	8 (22.9)	5 (14.3)	
Secondary/tertiary	27 (77.1)	30 (85.7)	
**Menopausal state**			0.63
Premenopausal	13 (37.1)	15 (42.9)	
Postmenopausal	22 (62.9)	20 (57.1)	
**BMI in kg/m^2^**			0.73
18.5–24.9 (normal)	14 (40.0)	11 (31.4)	
25.0–29.9 (overweight)	13 (37.1)	14 (40.0)	
≥30 (obese)	8 (22.9)	10 (28.6)	
**Median BMI (IQR)**	26.0 (22.7–29.2)	27.5 (23.4–32.4)	
**Skin complexion**			0.37
Black	7 (20.0)	7 (20.0)	
Dark brown	16 (45.7)	21 (60.0)	
Light	12 (34.3)	7 (20.0)	
**Alcohol intake**			0.61^b^
No	32 (91.4)	34 (97.1)	
Yes	3 (8.6)	1 (2.9)	
**Family history of cancer**			
No	27 (77.1)	33 (94.3)	0.04
Yes	8 (22.9)	1 (5.7)	
**Vitamin D categories**			0.09
Normal (≥75 nmol/L)	5 (14.3)	11 (31.5)	
Deficiency (<75 nmol/L)	30 (85.7)	24 (68.5)	

Abbreviations: BMI, body mass index (calculated as weight in kilograms divided by the square of height in metres).

a Values are given as mean ± SD, median (interquartile range) or number (percentage) unless stated otherwise.

b Fisher’s exact test.

**Table 2.** Characteristics of women with epithelial ovarian cancer (*n* = 35).

Characteristics	Number (%)
**Histological types of EOC**	
Serous cystadenocarcinoma	28 (80.0)
Mucinous cystadenocarcinoma	4 (11.4)
Endometrioid	2 (5.7)
Malignant Brenner	1 (2.9)
**Serum CA 125 Level**	
Normal levels (0–35 U/ml)	3 (8.6)
Abnormal levels (>35 U/ml)	32 (91.4)
**FIGO stage of the disease**	
Early stage	2 (5.7)
Advanced stage	33 (94.3)

Abbreviations: EOC, Epithelial ovarian cancer; CA 125, cancer antigen 125; FIGO, International Federation of Gynaecology and Obstetrics;

**Table 3.** Univariable logistic regression of risk factors of epithelial ovarian cancer.

Variables	OR	95% CI	*p*-value
**Age group (years)**			
<50	1.00	Reference	-
≥50	1.41	0.55–3.62	0.47
**BMI (kg/m^2^)**			
<25 (underweight/normal)	1.00	Reference	-
25-29 (overweight)	0.73	0.24–2.18	0.57
≥30 (Obese)	0.63	0.19–2.13	0.46
**Parity**			
0	1.00	Reference	-
1–4	0.87	0.23–3.30	0.84
>4	0.65	0.14–3.04	0.58
**Family history of cancer**			
No	1.00	Reference	-
Yes	4.89	0.96–24.97	0.06
**Alcohol use**			
No	1.00	Reference	-
Yes	3.19	0.32–32.24	0.33
**Educational qualification**			
At least primary education	1.00	Reference	-
At least secondary education	0.56	0.16–1.93	0.36
**Skin colour**			
Light	1.00	Reference	-
Black	0.58	0.14–2.37	0.45
Dark brown	0.44	0.14–1.38	0.16
**Coexisting morbidity**			
No	1.00	Reference	-
Yes	2.30	0.62–8.48	0.21
**Menopausal status**			
Premenopausal	1.00	Reference	-
Postmenopausal	1.27	0.49–3.31	0.63
**Vitamin D category**			
Normal (≥75 nmol/l)	1.00	Reference	-
Deficiency (<75 nmol/l)	2.75	0.84–9.00	0.09
**Serum vitamin D levels per 10 nmol/l**	0.96	0.93–0.99	0.04

Abbreviations: BMI, body mass index (calculated as weight in kilograms divided by the square of height in metres); OR, crude odds ratio.

**Table 4.** Multivariable conditional logistic regression of the major risk factors of epithelial ovarian cancer.

Variables	OR	(95% CI)	*p*-value
**Serum vitamin D levels per 10 nmol/L**	0.99	0.97–1.00	0.06
**Age (years)**			
<50	1.00	Reference	-
≥50	1.56	0.54–4.51	0.42
**Family history of cancer**			
No	1.00	Reference	-
Yes	5.88	1.05–32.94	0.04
**Skin colour**			
Light	1.00	Reference	-
Black	0.47	0.10–2.18	0.34
Dark brown	0.37	0.11–1.31	0.12

Abbreviations: CI, confidence interval; OR, adjusted odds ratio.
